# Determinants of Disease Phenotype Differences Caused by Closely-Related Isolates of Begomovirus Betasatellites Inoculated with the Same Species of Helper Virus

**DOI:** 10.3390/v7092853

**Published:** 2015-09-14

**Authors:** Jie Zhang, Mingqing Dang, Qingqing Huang, Yajuan Qian

**Affiliations:** 1 Institute of Biotechnology, Zhejiang University, Hangzhou 310058, China; jiezhang3553@163.com (J.Z.); dangmingqing2008@163.com (M.D.); huangqing_qing@126.com (Q.H.); 2 Institute of Plant Virology, Fujian Agriculture and Forestry University, Fuzhou 350002, China

**Keywords:** Tomato yellow leaf curl China virus, hybrid betasatellites, *βC1* gene, promoter

## Abstract

Tomato yellow leaf curl China virus (TYLCCNV) is a monopartite begomovirus associated with different betasatellites. In this study, we investigate two different isolates of Tomato yellow leaf curl China betasatellite (TYLCCNB) to determine what features of the viral genome are required for induction of characteristic phenotypic differences between closely-related betasatellite. When co-agroinoculated with TYLCCNV into *Nicotiana* spp. and tomato plants, TYLCCNB-Y25 induced only leaf curling on all hosts, while TYLCCNB-Y10 also induced enations, vein yellowing, and shoot distortions. Further assays showed that *βC1* of TYLCCNB-Y25 differs from that of TYLCCNB-Y10 in symptom induction and transcriptional modulating. Hybrid satellites were constructed in which the *βC1* gene or 200 nt partial promoter-like fragment upstream of the *βC1* were exchanged. Infectivity assays showed that a TYLCCNB-Y25 hybrid with the intact TYLCCNB-Y10 *βC1* gene was able to induce vein yellowing, shoot distortions, and a reduced size and number of enations. A TYLCCNB-Y10 hybrid with the intact TYLCCNB-Y25 *βC1* gene produced only leaf curling. In contrast, the TYLCCNB-Y25 and TYLCCNB-Y10 hybrids with swapped partial promoter-like regions had little effect on the phenotypes induced by wild-type betasatellites. Further experiments showed that the TYLCCNB-Y25 hybrid carrying the C-terminal region of TYLCCNB-Y10 *βC1* induced TYLCCNB-Y10-like symptoms. These findings indicate that the βC1 protein is the major symptom determinant and that the C-terminal region of *βC1* plays an important role in symptom induction.

## 1. Introduction

Geminiviruses (family *Geminiviridae*) are a group of plant viruses with single-stranded DNA genomes packaged in distinctive twinned particles. Based on the genome structure, insect vectors, and host range, *Geminiviridae* have been classified into seven genera *Begomovirus*, *Mastrevirus*, *Curtovirus*, *Topocuvirus*, *Becurtovirus*, *Turncurtovirus*, and *Eragrovirus* [1], with the majority of described geminiviruses belonging to the genus *Begomovirus*. Begomoviruses have either bipartite or monopartite single-stranded DNA genomes [2], and the majority of monopartite begomoviruses are reported to be associated with betasatellites [3,4]. All of the characterized betasatellites are circular, single-stranded DNA molecules approximately half the size of their helper begomoviruses (~1.3 kb). The betasatellites share little, if any, sequence identity with their viral DNA, and are dependent on their helper viruses for replication, encapsidation, insect transmission, and movement in plants [3]. Sequence alignments showed that the reported betasatellites contain three common features: a satellite conserved region (SCR) adjacent to a putative stem-loop structure containing the nonanucleotide 5′-TAATATTAC-3′, a conserved complementary-sense gene (*βC1*), and an A-rich region located upstream of the *βC1* gene that contributes to the size requirements for virus encapsidation and/or virus movement [5]. Using both transient expression and stable transformation systems, βC1 has been identified as a pathogenicity determinant as well as a suppressor of RNA silencing [6,7,8,9,10].

Tomato yellow leaf curl China virus (TYLCCNV) is a typical monopartite begomovirus identified to be associated with a betasatellite, which has been found in tobacco, tomato, *Siegesbeckia orientalis*, and kidney bean (*Phaseolus vulgaris*) plants in China [6,11,12]. Infectivity assays showed that TYLCCNV requires a betasatellite for induction of disease symptoms in host plants [4,6]. It is worth noting that different TYLCCNV/TYLCCNB (Tomato yellow leaf curl China betasatellite) disease complexes often produce distinct symptom phenotypes in their natural hosts. The contributions of different isolates of TYLCCNV and the betasatellite to symptom production are not very clear. Previously, Ding *et al*. [13] reported that the βC1 protein was the main determinant of symptom differences between TYLCCNV/TYLCCNB and Tobacco curly shoot virus (TbCSV)/Tobacco curly shoot betasatellite (TbCSB). In this study, we investigate two different isolates of TYLCCNB to determine what features of the viral genome are required for induction of characteristic disease symptoms between closely related betasatellite isolates together with the same species of helper virus. Our findings demonstrate that the βC1 protein is the major symptom determinant and the C-terminal region of *βC1* plays an important role in symptom induction.

## 2. Results

### 2.1. Infectivity of TYLCCNV and its Associated Betasatellite

**Figure 1:**
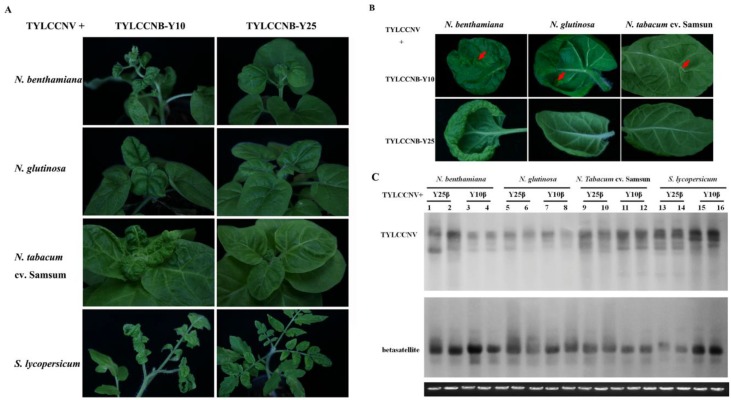
Symptoms induced by co-inoculation with TYLCCNV with TYLCCNB-Y10 or TYLCCNB-Y25 satellites in *N. benthamiana*, *N. glutinosa*, *N. tabacum* cv. Samsun and *S. lycopersicum*. (**A**) Whole plants. Photographs of plants were taken at 30 days post inoculation (dpi); (**B**) abaxial surfaces of leaves. Arrowheads indicate enations; (**C**) Southern blot analysis for viral DNA in *N. benthamiana*, *N. glutinosa*, *N. tabacum* cv. Samsun, and *S. lycopersicum* plants agroinoculated with TYLCCNV along with TYLCCNB-Y10 or TYLCCNB-Y25. The blots were probed either for TYLCCNV (top) or for betasatellite (bottom). The lower panel represents an ethidium bromide-stained gel of DNA samples as a loading control. Y10β, TYLCCNB-Y10; Y25β, TYLCCNB-Y25.

Two related betasatellite isolates, TYLCCNB-Y10 (GenBank accession no. AJ421621) and TYLCCNB-Y25 (AJ421619) associated with TYLCCNV share 86.0% nucleotide sequence identity, while their *βC1* genes share 89.8% amino acid identity [4]. Generally, begomovirus betasatellite components with sequence identity below the threshold value of 78% are considered to be distinct betasatellite species [2]. According to the above criteria, the two betasatellites are considered to be different isolates of TYLCCNB. Previously, TYLCCNB-Y10 was shown to be indispensable for symptom induction [4,6]. To further understand the contributions of different isolates of TYLCCNB to disease phenotypes, infectious clones of TYLCCNB-Y10 or TYLCCNB-Y25 were co-inoculated together with TYLCCNV into *Nicotiana benthamiana*, *N. glutinosa*, *N. tabacum* cv. Samsun, and *Solanum lycopersicum* plants (Table 1). Consistent with the previous studies [4,6], in the presence of TYLCCNB-Y10, TYLCCNV induced severe leaf curling and vein yellowing in all four host plants, and also shoot distortion in *N. benthamiana* (Figure 1A) At the later stages of infection, enations could be observed in *Nicotiana* spp. (Figure 1B) and *S. lycopersicum* (data not shown), with the highest number of enations present on *N. benthamiana* (Table 1). In contrast, when TYLCCNV was co-inoculated with TYLCCNB-Y25, only leaf curling was observed in all four hosts (Figure 1A,B). Southern blot hybridization analysis showed that both the helper virus and betasatellite were present in systemically infected leaves of *N. benthamiana*, *N. glutinosa*, *N. tabacum* cv. Samsun, and *S. lycopersicum* plants (Figure 1C). These results suggest that the vein yellowing, shoot distortion, and enation phenotypes co-segregate with TYLCCNB-Y10.

### 2.2. Phenotypes of Transgenic Plants Expressing TYLCCNB-Y25 βC1

**Figure 2:**
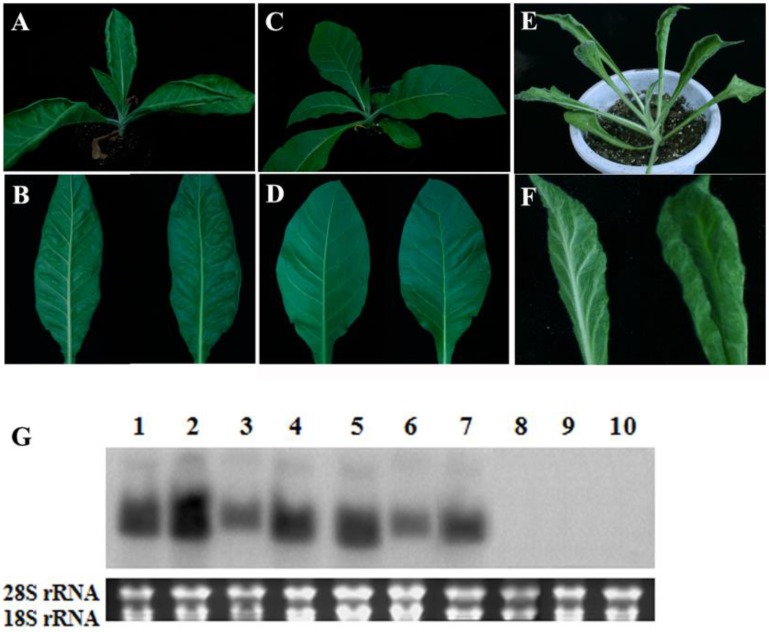
Phenotypes and Northern blot analysis of transgenic plants containing the TYLCCNB *βC1* gene. Phenotypes of transgenic tobacco transformed with 35S-Y25βC1, symptomatic plants (**A** and **B**); symptomless (**C** and **D**); 35S-Y10βC1 (**E** and **F**); (**G**) Northern blot analysis. Lanes 1–4, 35S-Y10βC1; lanes 5–7, 35S-Y25βC1 (symptomatic plants); lanes 8,9, 35S-Y25βC1 (symptomless plants); lane 10, pCHF3 empty vector. The ethidium bromide-stained gel shown below the blot indicates equal loading of total RNA.

A previous study showed that transgenic *N*. *tabacum* and *N*. *benthamiana* plants expressing TYLCCNB-Y10 βC1 displayed a range of disease symptoms similar to those of virus-infected tobacco plants, such as leaf distortion, severe leaf curling, blistering or leaf protuberances (Figure 2E,F) [6]. To determine whether the *βC1* gene of TYLCCNB-Y25 is also responsible for symptom induction, transgenic *N. tabacum* plants expressing TYLCCNB-Y25 βC1 were generated via *Agrobacterium*-mediated transformation. Twenty-five positive seedlings were chosen by PCR analysis using primers specific for TYLCCNB-Y25 *βC1*. Of the positive transgenic seedlings, only three developed virus-infection-like symptoms, including upward leaf curling, and interveinal blistering of leaves (Figure 2A,B), while the remaining seedlings developed normally and remained symptomless (Figure 2C,D). Compared with the phenotypes induced by TYLCCNB-Y10 βC1 overexpression in transgenic seedlings, those induced by overexpression of TYLCCNB-Y25 βC1 were mild. In order to further determine the expression level of *βC1* in transgenic seedlings, Northern blot analysis was performed by hybridization with a TYLCCNB-Y25 *βC1* probe. As shown in Figure 2G, both TYLCCNB-Y10 and TYLCCNB-Y25 *βC1* transgenic plants with abnormal phenotypes displayed comparable blot intensities, while the transgenic plants with symptomless phenotypes failed to accumulate detectable amounts of *βC1* transcript. These data indicated that the *βC1* gene of TYLCCNB-Y25 is capable for producing disease symptoms, but differs from that of TYLCCNB-Y10 in symptom induction when constitutively expressed in transgenic plants.

### 2.3. Analysis of the Putative Promoter of TYLCCNB-Y25

**Figure 3:**
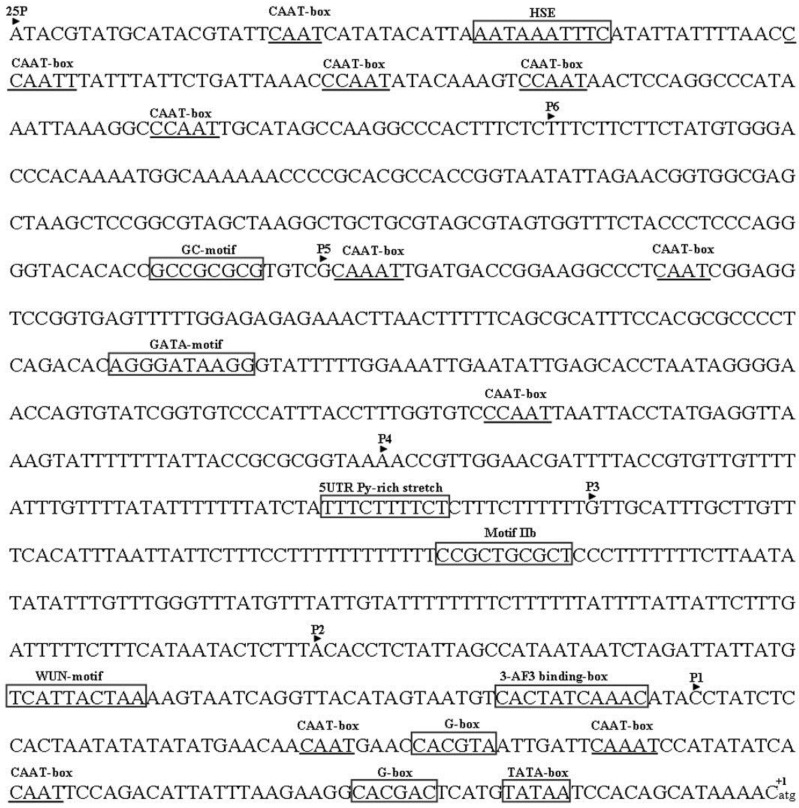
The nucleotide sequence encompassing the entire non-coding region (982 nt) upstream of the TYLCCNB-Y25 *βC1* open reading frame. The translation start site A is labeled +1. The putative motifs are shown in frame or marked by underline.

A previous study showed that the TYLCCNB-Y10 *βC1* promoter had only 13% of Cauliflower mosaic viru*s* (CaMV) 35S promoter activity [14]. The sequence of the putative promoter encompassing the entire non-coding region (982 nt) upstream of the TYLCCNB-Y25 *βC1* open reading frame was analyzed using the PlantCARE database (Figure 3). A typical TATA box was identified to locate 20 nt upstream of the transcription start site (-20 nt). The CAAT box was found at sites -56 nt, -71 nt, -92 nt, -491 nt, -650 nt, -673 nt, -857nt, -887 nt, -901 nt, -925 nt, and -963 nt. A number of putative regulatory motifs and *cis*-elements were also predicted, including a HSE sequence (at site -948 nt), a GC-motif (at site -686 nt), a GATA-motif (at site -575 nt), a motif IIb (at site -316 nt), a WUN-motif (at site -170 nt), and a AF3 binding-box (at site -135 nt) . The G-box and 5UTR Py-rich stretch that had been shown to be essential for the activity of the geminivirus promoters were found to locate at sites -31 nt, -84 nt, and -386 nt [15,16].

**Figure 4:**
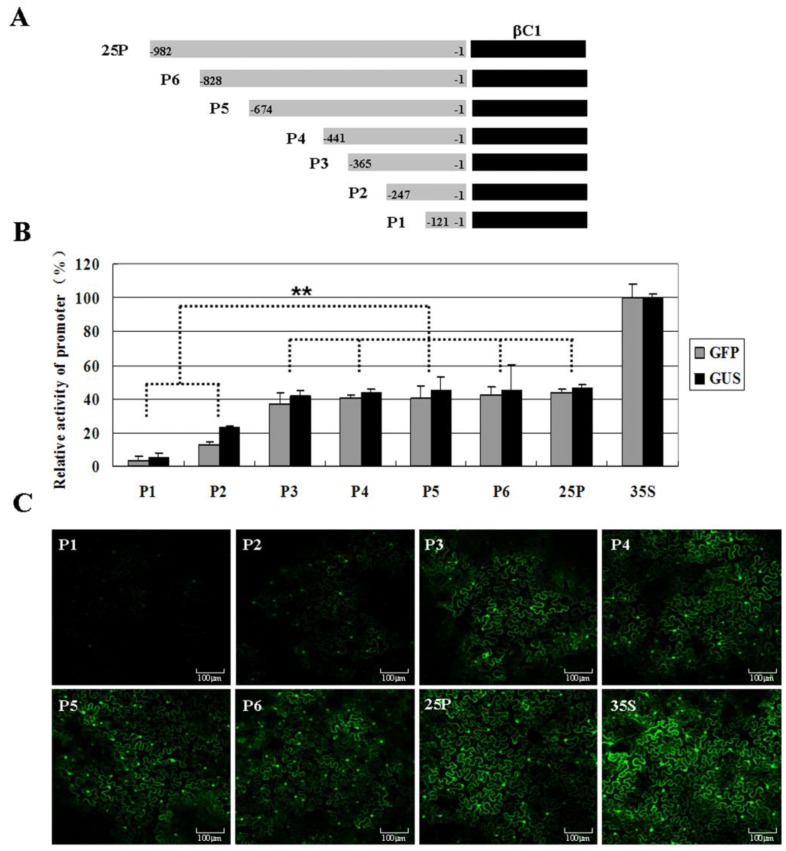
Identification of promoter activity in *N*. *benthamiana* leaves after transiently expressing various TYLCCNB-Y25 *βC1*-derived promoters. (**A**) Schematic representation of the TYLCCNB-Y25 genome and various *βC1* promoters fused to a promoter-less pINT121 or pCHF3:GFP vector; (**B**) fluorometric activity analysis after transient expression various promoter constructs. The mean GUS or GFP activity from the CaMV 35S promoter of pINT121 or pCHF3:GFP was considered as 100% and used to standardize the activities for all of the other constructs, respectively. Columns represent the mean value, with standard error of the mean. The significant difference between treatments (** *p* ≤ 0.01) was shown; (**C**) confocal microscopy showed GFP fluorescence after transient expression various promoter constructs.

To determine the promoter of TYLCCNB-Y25 *βC1* and the *cis*-elements responsible for the transcriptional control of TYLCCNB-Y25 *βC1*, the entire upstream, non-coding region (982 nt) of *βC1* and series of deletions within the region were constructed and fused to β-glucuronidase (*GUS*) or green fluorescent protein (*GFP*) reporter gene (Figure 4A). These promoter constructs were agro-inoculated into *N. benthamiana* leaves. Fluorometric GUS assays revealed that the longest promoter (25P) displayed about 46.6% of the promoter activity of the CaMV 35S promoter (Figure 4B). Deletion of the region from -982 to -366 (P3) made no significant differences in GUS activity compared with p25 (*p* > 0.05). Interestingly, deletion of the region from -982 to -248 nt (P2) resulted in a marked reduction in GUS expression level to just 23.3% that of CaMV 35S promoter, which was significantly different from that of 25P (*p* < 0.01). Deletion from -982 to -122 nt (P1) declined the promoter activity to 5.5% of that driven by the CaMV 35S promoter. On the other hand, calculation of GFP fluorescence intensity as well as fluorescent image captured by confocal microscope indicated 25P and P3 produced relatively higher levels of fluorescence, but much weaker than the positive control pCHF3:GFP (Figure 4B,C). A mark reduction in GFP expression level driven by P2 was observed. Fluorometric GFP assays also revealed the sequence within a 247 nt region upstream of the TYLCCNB-Y25 *βC1* was basic for *βC1* promoter activity. These results were consistent with those of the fluorometric GUS assay.

### 2.4. Infectivity and Symptoms of Hybrid Betasatellites

**Figure 5:**
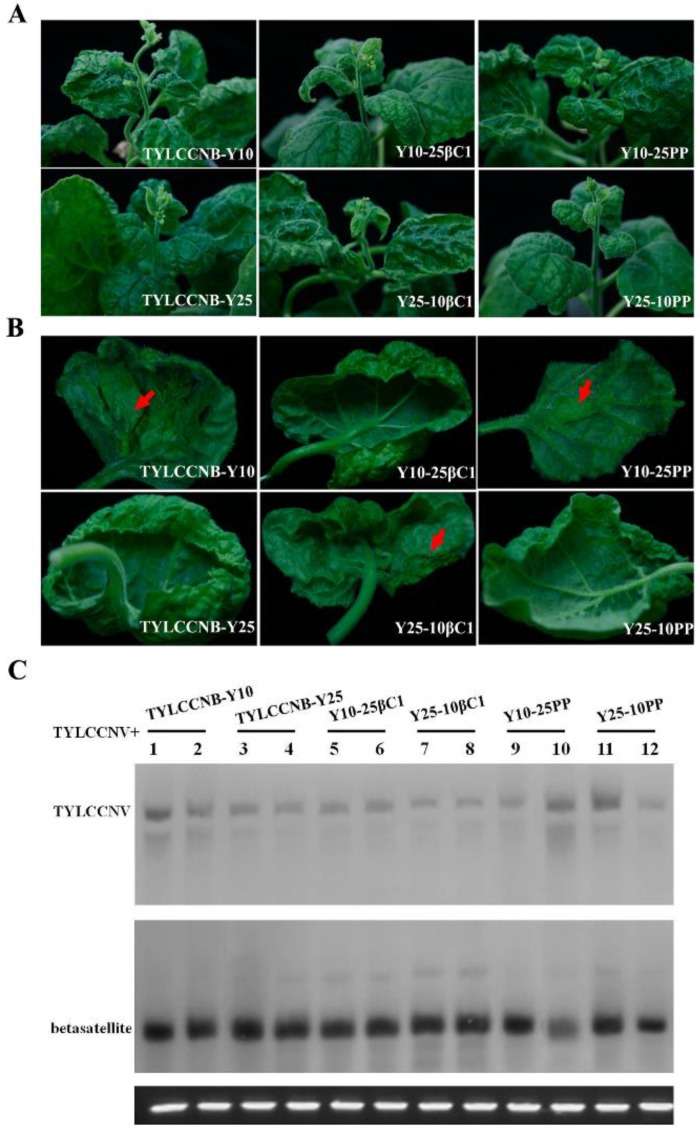
Symptoms induced by co-inoculation with TYLCCNV and either wild-type or hybrid satellites of TYLCCNB-Y10 and TYLCCNB-Y25 in *N. benthamiana*. (**A**) Whole plants. Photographs of plants were taken at 30 dpi; (**B**) abaxial surfaces of leaves. Arrowheads indicate enations; (**C**) Southern blot analysis for viral DNA in *N. benthamiana* plants. The blots were probed either for TYLCCNV (top) or for betasatellite (bottom). The lower panel represents an ethidium bromide-stained gel of DNA samples as a loading control.

To understand the roles of betasatellite-encoded βC1 and the putative *βC1* gene promoter in symptom production, the *βC1* gene or the 200 nt fragment upstream of the translation start site of *βC1* (referred as PP) were precisely exchanged between TYLCCNB-Y10 and TYLCCNB-Y25 by an overlap-extension PCR method. The infectious constructs, Y10-25βC1, Y10-25PP, Y25-10βC1, and Y25-10PP were individually co-inoculated with TYLCCNV into *N. benthamiana* plants. PCR detection and symptom observation showed that all the betasatellite hybrids could systemically infect *N. benthamiana* with a high efficiency (Table 1). Interestingly, the symptoms induced by Y25-10βC1 or Y10-25PP together with TYLCCNV consisted of leaf curling, vein yellowing, and shoot distortion, as well as enations, which were similar to those induced by wild-type TYLCCNB-Y10 except that the onset of the enations were delayed and they were smaller. It is worth noting that Y10-25βC1 or Y25-10PP together with TYLCCNV elicited TYLCCNB-Y25-like symptoms and produced only leaf curling (Table 1, Figure 5A,B). These results indicated that the *βC1* gene from TYLCCNB-Y10 plays an essential role in producing typical symptoms, including vein yellowing, shoot distortion, and enations, and suggested that the putative promoter region of TYLCCNB *βC1* had little effect on the phenotypes induced by TYLCCNV plus TYLCCNB.

The accumulation of viral DNA and hybrid betasatellite DNA in systemically-infected leaf tissues of *N. benthamiana* was determined by Southern blot hybridization. The results showed that the levels of helper virus were similar, irrespective of which particular betasatellite was present (Figure 5C). Additionally, the hybrid betasatellites Y10-25βC1, Y10-25PP, Y25-10βC1, and Y25-10PP were efficiently trans-replicated by TYLCCNV and displayed similar accumulation levels. The levels of viral and hybrid betasatellite DNA were not obviously related to the previously described symptom differences.

**Table 1:** Symptoms induced by wild-type and hybrid betasatellites of TYLCCNB-Y10 and TYLCCNB-Y25 co-infected with TYLCCNV.

Inoculum (TYLCCNV+)	Plant Species	Symptoms ^a^	Infectivity ^b^
TYLCCNB-Y10	*N. benthamiana*	LC, YV, EN, SD	35/35(34)
	*N. glutinosa*	LC, YV, EN	14/15(5)
	*N.* *tabacum* cv. Samsun	LC, YV, EN	12/15(5)
	*S. lycopersicum*	LC, YV, EN	8/14(3)
TYLCCNB-Y25	*N. benthamiana*	LC	34/35
	*N. glutinosa*	LC	15/15
	*N.* *tabacum* cv. Samsun	LC	9/15
	*S. lycopersicum*	LC	7/15
Y25-10βC1	*N. benthamiana*	YV, LC, SD, EN	30/30(30)
Y10-25βC1	*N. benthamiana*	LC	29/30
Y25-10PP	*N. benthamiana*	LC	20/25
Y10-25PP	*N. benthamiana*	MYV, LC, SD, EN	25/25(25)
Y10-25CβC1	*N. benthamiana*	LC	28/28
Y10-25MβC1	*N. benthamiana*	MYV, LC, SD, EN	28/28(20)
Y10-25NβC1	*N. benthamiana*	MYV, LC, SD, EN	22/28(12)
Y25-10CβC1	*N. benthamiana*	MYV, LC, SD, EN	27/28(15)
Y25-10MβC1	*N. benthamiana*	LC	28/28
Y25-10NβC1	*N. benthamiana*	LC	24/28

^a^ LC, leaf curling; YV, yellow vein; MYV, mild yellow vein; EN, enations; SD, shoot distortion; ^b^ No. of infected plants/no. of inoculated plants (no. of enations) (total of three independent trials).

### 2.5. Minor Determinants of Symptoms within the TYLCCNB βC1 Gene

**Figure 6:**
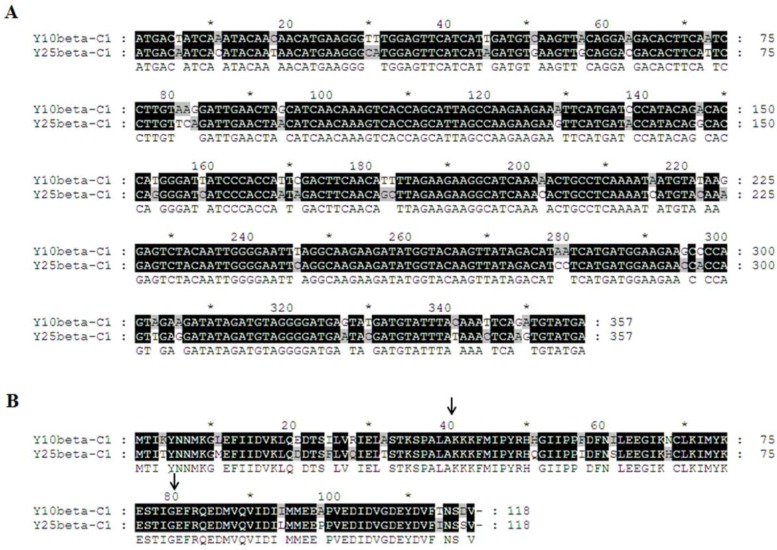
Alignment of *βC1* nucleotide sequence (**A**) and predicted βC1 amino acid sequence (**B**) from TYLCCNB-Y10 and TYLCCNB-Y25. Arrowheads indicate the split sites.

**Figure 7:**
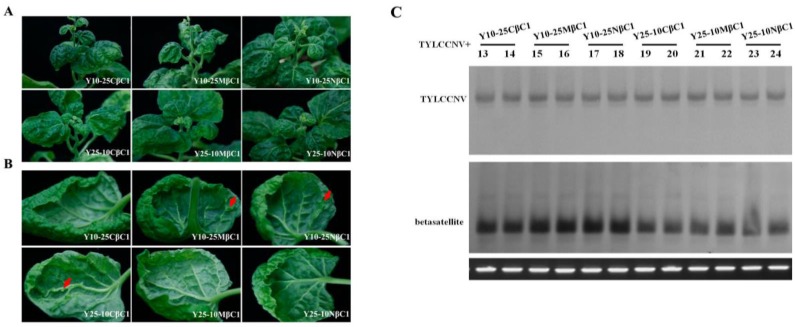
Symptoms induced by co-inoculation with TYLCCNV with hybrid betasatellites of TYLCCNB-Y10 and TYLCCNB-Y25 in *N. benthamiana*. (**A**) Whole plants. Photographs of plants were taken at 30 dpi; (**B**) abaxial leaf surfaces. Arrowheads indicate enations; (**C**) Southern blot analysis for viral DNA in *N. benthamiana* plants. The blots were probed either for TYLCCNV (top) or for betasatellite (bottom). The lower panel represents an ethidium bromide-stained gel of DNA samples as a loading control.

Sequence analysis showed that TYLCCNB-Y10 *βC1* and TYLCCNB-Y25 *βC1* shared more than 89.8% amino acid (aa) identity using Clustal W method in MegAlign (Lasergene), with 14 out of 118 aa showing differences (Figure 6). To further map the symptom determinants within the βC1 protein, viable hybrid betasatellites (designated Y10-25CβC1, Y10-25MβC1, Y10-25NβC1, Y25-10CβC1, Y25-10MβC1, and Y25-10NβC1) were obtained by exchanging the N-terminal (between 1 to 40 aa), middle (between 41 to 80 aa) or C-terminal region (between 81 to 118 aa) between TYLCCNB-Y10 *βC1* and TYLCCNB-Y25 *βC1*. Using TYLCCNV as helper virus, these infectious clones of hybrid TYLCCNB-Y10 or TYLCCNB-Y25 betasatellites were inoculated into *N. benthamiana* plants. Y25-10CβC1 elicited similar symptoms to TYLCCNB-Y10, including leaf curling, shoot distortions, mild vein yellowing and enations, although the enations were smaller and their numbers were reduced (Table 1, Figure 7A,B). Interestingly, Y25-10MβC1 and Y25-10NβC1 elicited TYLCCNB-Y25-like symptoms. Y10-25CβC1 produced only a leaf curling phenotype similar to TYLCCNB-Y25, while the symptoms induced by Y10-25MβC1 and Y10-25NβC1 were TYLCCNB-Y10-like (Table 1, Figure 7A,B). Southern blot hybridization analysis showed that there were no significant differences among all the inoculums for both the helper virus and betasatellite (Figure 7C), indicating that neither the accumulation of viral nor hybrid betasatellite DNA was related to the observed symptom differences. Taken together, the above results suggested that the C-terminal region of TYLCCNB *βC1* plays an important role in symptom induction, but that the other parts of the gene can also play a role in symptom production.

## 3. Discussion

Begomovirus/betasatellite disease complexes have emerged as major pathogens of crops worldwide, and they induce characteristic phenotypes including leaf curling, vein yellowing, shoot distortion, and enations in host plants [17,18]. Comparisons of the reported betasatellites have shown that the position and size of the *βC1* ORF is conserved [4]. Evidence for the involvement of betasatellites in modulation of symptom expression has been provided previously [4,5,8,19]. In addition, mutagenesis of betasatellites has shown that βC1 is required for disease symptom induction. In this study, to further investigate the role of betasatellites in pathogenicity, two different isolates of TYLCCNB were investigated to determine what features of the viral genome are required for induction of characteristic disease symptoms.

Previous studies have shown that betasatellites can be trans-replicated stably by a non-cognate helper begomovirus [13,20,21], suggesting that the interaction between begomoviruses and betasatellites is not highly specific. Pseudorecombination has been used to localize a genetic determinant of host adaptation, tissue tropism, and host range in bipartite geminiviruses [22,23,24]. An alternative strategy, which is more generally applicable, is to construct recombinant hybrid viruses. Recently, these approaches have been applied to the analysis of two well-characterized begomovirus/betasatellite disease complexes-TbCSV/TbCSB and TYLCCNV/TYLCCNB in tobacco and tomato plants. Using the criteria of symptom severity and the extent of viral DNA accumulation, Ding *et al*. [13] found that the symptom differences between TbCSV/TbCSB and TYLCCNV/TYLCCNB were determined by the betasatellites, and that the βC1 protein was the symptom determinant. In this study, the begomovirus/betasatellite pairs TYLCCNV/TYLCCNB-Y10 and TYLCCNV/TYLCCNB -Y25 were co-inoculated into tobacco and tomato plants and found to induce distinct symptom phenotypes, suggesting that a determinant of characteristic symptoms is also localized on the betasatellite.

Virus-like phenotypes in tobacco plants expressing *βC1* were observed, either under the control of the CaMV 35S promoter or the putative promoter present in the betasatellite itself [8,9,25,26]. Previously Cui *et al*. [6] showed that transgenic expression of the *βC1* gene of TYLCCNB-Y10 caused leaf distortion, interveinal protuberances, or small interveinal tissue outgrowths on the undersides of some leaves of transgenic *N. tabacum*. In addition, transgenic Arabidopsis plants expressing the *βC1* gene from TYLCCNB displayed upward leaf curl, bending shoots, as well as enations from the abaxial leaf surfaces [27]. Here, when transgenic tobacco plants expressed the *βC1* gene from TYLCCNB-Y25, abnormal phenotypes like upward leaf curling and interveinal blistering of leaves were also observed. The symptoms developed by TYLCCNB-Y10 βC1 transgenic plants were obviously more severe than those exhibited by expression of TYLCCNB-Y25 βC1. These results in the transgenic system indicated that TYLCCNB-Y25 βC1 has relatively weak virulence compared with TYLCCNB-Y10 βC1.

In order to understand the role of βC1 in determining viral symptoms, a set of hybrids were constructed to precisely swap the *βC1* gene between TYLCCNB-Y10 and TYLCCNB-Y25. The phenotypes of a given Y25-10βC1 hybrid betasatellite represented the effects of TYLCCNB-Y10-like symptom-specific gain of function resulting from the TYLCCNB-Y10 *βC1* sequences it contains. The hybrid betasatellite Y10-25βC1 produced leaf curling symptoms, a TYLCCNB-Y25-like characteristic phenotype, and resulted from insertion of the TYLCCNB-Y25 *βC1* sequence. Taken together, the above results showed that the betasatellite-encoded βC1 protein is the genetic determinant for the symptom differences observed between the TYLCCNV/TYLCCNB-Y10 and TYLCCNV/TYLCCNB-Y25 disease complexes. To our knowledge, this is the first description of the genetic determinant of the phenotypic differences between closely related betasatellite isolates together with the same species of helper virus.

Sequence analysis showed that the two putative promoters encompassing the entire non-coding region upstream of the TYLCCNB-Y10 and TYLCCNB-Y25 *βC1* share 84.8% sequence identity. A previous study showed that base substitutions in a promoter-like region affected the host range and symptoms of Maize streak virus [28]. Similarly, Petty *et al*. [24] demonstrated that host-adaptation of Tomato golden mosaic virus and Bean golden mosaic virus was determined cooperatively by coding and noncoding sequences in the genome. To further elucidate additional sequences important for modulating viral symptoms, we constructed hybrid TYLCCNB-based betasatellites in which the partial promoter regions were precisely swapped. The hybrid Y10-25PP produced TYLCCNB-Y10-like symptoms; however, the onset of the appearance of enations was delayed, and they were reduced in size. Thus, we concluded that the promoter of the *βC1* gene associated with closely related isolates of betasatellite has little influence on disease symptom production.

In an effort to understand what features of the *βC1* gene are required for the production of characteristic disease symptoms, we looked for gain-of-function phenotypes generated by TYLCCNB-based hybrid betasatellites. Like wild-type TYLCCNB-Y10, the hybrid Y25-10CβC1 could cause leaf curling, shoot distortions, mild vein yellowing, as well as enations, whereas the hybrid Y10-25CβC1 produced only leaf curling, similar to TYLCCNB-Y25. Our infection assay using hybrid betasatellites showed that the C-terminal part of TYLCCNB-Y10 *βC1* plays an important role in characteristic symptom induction. Interestingly, previous reports showed that in the presence of Cotton leaf curl Multan virus (CLCuMV), betasatellite from cotton leaf curl disease (CLCuD) from Pakistan (CLCDβΔ01-Pak) encoding an N-terminally truncated form of the βC1 protein could produce typical CLCuD symptoms, while betasatellite from Hibiscus leaf curl disease (HLCDβ01-Pak) containing a C-terminal truncation of the βC1 protein was not infectious in cotton [3]. Similarly, Saeed *et al*. [9] also showed that expression of the N-terminal 40 amino acids of CLCuB βC1 was not sufficient for its pathogenicity. It remains unclear how the C-terminal region of betasatellite βC1 is involved in regulating symptom production. A possible explanation for this is that exchange of the region encoding the C-terminal region of βC1 between closely related betasatellite isolates might affect its interaction with a host protein and/or its function as a RNA-silencing suppressor. A number of plant viruses have been reported to encode suppressors of RNA-induced gene silencing as a counter-defense strategy [29,30]. The C4 protein of Tomato leaf curl virus-Australia (ToLCV) has been identified to be a suppressor of RNA gene silencing. Evidence has shown that a 12 aa region in the C-terminal region of ToLCV C4 is not only required for suppression of gene silencing but is also essential for its binding to a novel shaggy-like kinase (SlSK) and the expression of disease symptoms [31]. TYLCCNB-Y10 βC1 has been shown to be a suppressor of PTGS and TGS [6,10]. Previous studies showed that TYLCCNB-Y10 βC1 interacted with ASYMMETRIC LEAVES1 to alter leaf development and suppress selected jasmonic acid responses [27]. Shen *et al*. [32] reported that a tomato SUCROSE-NONFERMENTING1-related kinase, designated SlSnRK1, attenuated geminivirus infection by interacting with and phosphorylating the TYLCCNB-Y10 βC1 protein. Sequence alignment identified four amino acid differences between the C-terminal region of the TYLCCNB-Y10 and TYLCCNB-Y25 *βC1* genes. Further investigations will be necessary to elucidate whether amino acid divergence impacts the function of TYLCCNB βC1 as a pathogenicity determinant and RNA-silencing suppressor, and whether or not it mediates its interaction with host protein(s) resulting in viral symptom modulation.

## 4. Materials and Methods

### 4.1. Construction of Infectious Clones of Wild-Type and Hybrid Betasatellites

**Figure 8:**
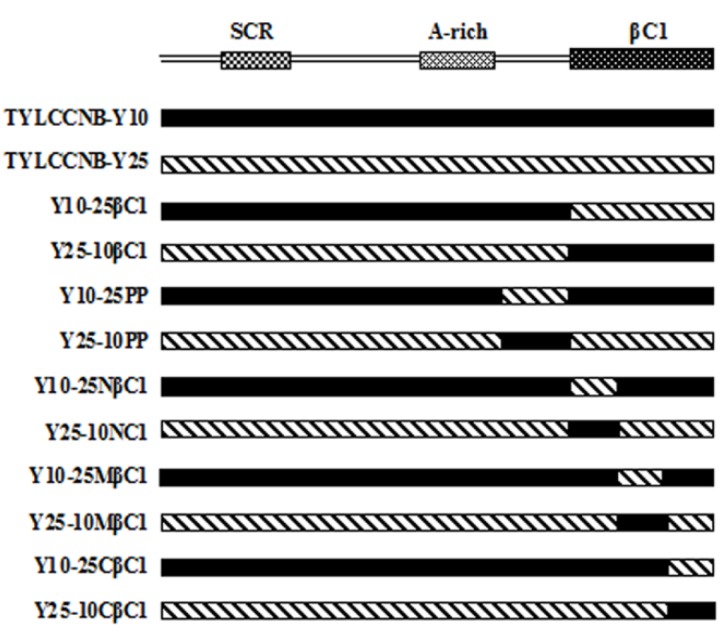
Schematic representation showing the organization of hybrid betasatellites. Betasatellite organization is shown as linear DNA in the complementary sense. SCR, satellite conserved region; A-rich, adenine-rich; βC1, *βC1* gene; TYLCCNB-Y10, tomato yellow leaf curl China betasatellite-Y10; TYLCCNB-Y25, tomato yellow leaf curl China betasatellite-Y25.

Plasmids containing hybrid betasatellite components were constructed by a splicing overlap-extension PCR strategy as described previously [13]. Briefly, hybrid betasatellites containing an exchangeable *βC1* ORF or putative partial promoter (hereafter abbreviated PP, approximately 200 nt upstream of the *βC1* gene) were obtained in a single molecule by three independent PCRs, using the full-length clones of TYLCCNB-Y10 and TYLCCNB-Y25 amplified with the universal primer pair β01/β02 [33] as templates (Figure 8). The overlapping PCR products were inserted into a pGEM-T easy vector (Promega, Madison, WI, USA) to produce the following clones: pGEM-Y25-10βC1 (in which the *βC1* gene of TYLCCNB-Y25 was substituted by the TYLCCNB-Y10 *βC1* gene), pGEM-Y10-25βC1 (in which the *βC1* gene of TYLCCNB-Y10 was substituted by the TYLCCNB-Y25 *βC1* gene); pGEM-Y25-10PP (in which the 200 nt fragment upstream of the translation start site of TYLCCNB-Y25 *βC1* was substituted by that of TYLCCNB-Y10); pGEM-Y10-25PP (in which the 200 nt fragment upstream of translation start site of the TYLCCNB-Y10 *βC1* gene was substituted by that of TYLCCNB-Y25); pGEM-Y25-10CβC1 (in which the C-terminal region of the *βC1* ORF of TYLCCNB-Y25 was substituted by that of TYLCCNB-Y10); pGEM-Y10-25CβC1 (in which the C-terminal region of the *βC1* ORF of TYLCCNB-Y10 was substituted by that of TYLCCNB-Y25); pGEM-Y25-10MβC1 (in which the middle part of the *βC1* ORF of TYLCCNB-Y25 was substituted by that of TYLCCNB-Y10); pGEM-Y10-25MβC1 (in which the middle part of the *βC1* ORF of TYLCCNB-Y10 was substituted by that of TYLCCNB-Y25); pGEM-Y25-10NβC1 (in which the N-terminal region of the *βC1* ORF of TYLCCNB-Y25 was substituted by that of TYLCCNB-Y10); and pGEM-Y10-25NβC1 (in which the N-terminal region of the *βC1* ORF of TYLCCNB-Y10 was substituted by that of TYLCCNB-Y25). All clones were completely sequenced to confirm that no mutations were introduced by PCR. Dimeric constructs of hybrid betasatellite clones for agroinoculation, pBIN-Y25-10βC1, pBIN-Y10-25βC1, pBIN-Y25-10PP, pBIN-Y10-25PP, pBIN-Y25-10CβC1, pBIN-Y10-25CβC1, pBIN-Y25-10MβC1, pBIN-Y10-25MβC1, pBIN-Y25-10NβC1, pBIN-Y10-25NβC1, together with infectious clones of a tandem repeat of TYLCCNB-Y25 (pBIN-2Y25), were produced using the method described by Zhou *et al*. [4]. The construction of infectious clones of TYLCCNV isolate Y10 and TYLCCNB-Y10 have been described previously [6].

### 4.2. Construction of Promoter Expression Vectors

The putative promoter region of the *βC1* gene, were amplified with a series of primers (Supplementary Table S1), using the TYLCCNB-Y25 infectious clone as template. The amplified fragments were cloned into the pGEM-T Easy vector for sequencing, and then digested individually with *Hin*dIII and *Bam*HI or *Eco*RI*/Sac*I restriction enzymes after sequencing. The resulting fragments were inserted between the *Hin*dIII and *Bam*HI sites in the binary vector pINT121 or *Eco*RI*/Sac*I sites in the binary vector pCHF3:GFP respectively [34] to replace the CaMV 35S promoter, producing various expression constructs: pINT-25P, pINT-P1, pINT-P2, pINT-P3, pINT-P4, pINT-P5, pINT-P6 pCHF3-25P, pCHF3-P1, pCHF3-P2, pCHF3-P3, pCHF3-P4, pCHF3-P5, and pCHF3-P6 (Figure 4A).

### 4.3. Construction of the TYLCCNB-Y25 βC1 Transgenic Expression Vector

The entire TYLCCNB-Y25 *βC1* gene (357 nt) was amplified with the Y25βC1-F/Y25βC1-R primer pair (Supplementary Table S1) using the TYLCCNB-Y25 infectious clone as the template. The amplified fragment was cloned into pGEM-T Easy and then digested with *Bam*HI and *Sal*I after sequencing. The resulting fragment was inserted into the *Bam*HI and *Sal*I sites between the CaMV 35S promoter and the nopaline synthase (nos) terminator in the expression vector pCHF3 to produce 35S-Y25βC1. The 35S-Y10βC1 construct was described previously [6].

All DNA constructs were introduced individually into *Agrobacterium tumefaciens* strain EHA105 by electroporation.

### 4.4. Agroinoculation of Plants

*A. tumefaciens* cultures harboring different constructs were incubated in YEP medium (Yeast extract/tryptone medium) at 28 °C overnight, and infiltrated into leaves of plants at the six-leaf stage using a 1 ml plastic syringe. For the co-inoculation of infectious virus clones, equal amounts of agrobacterium cultures carrying the helper virus and betasatellite were mixed to inoculate plants of *N.* *benthamiana*, *N. glutinosa*, *N. tabacum*, and *S.* *lycopersicum*. The inoculated plants were grown in greenhouse at 25 °C under a 16 h light/8 h dark cycle and observed daily for symptom development.

### 4.5. Plant Transformation

*A. tumefaciens* cultures harboring expression vectors were used to transform leaf explants of *N. tabacum* as previously described [35]. Transformants were selected on MS medium (Murashige and Skoog medium, Duchefa, Haarlem, The Netherlands) containing 100 μg/mL kanamycin and 500 μg/mL carbenicillin. Regenerated kanamycin-resistant shootlets were grown on a rooting medium and then transferred to soil after confirmation of the presence of the transgene by PCR.

### 4.6. Analysis of DNAs in Inoculated Plants or RNAs in Transformed Plants

Total DNA extracts from leaf tissues were isolated at 30 days post-inoculation (dpi) as described by Xie *et al*. [36], fractioned on a 1% agarose gel, and transferred to Hybond-N^+^ membranes (GE Healthcare, Piscataway, NJ, USA) by capillary blotting. Membranes were hybridized separately to DIG-labeled probes of the *CP* gene from the helper virus and the full-length TYLCCNB-Y10 betasatellite, using the DIG High Prime DNA Labeling and Detection Starter Kit I, according to the manufacturer’s instructions (Roche Diagnostics GmbH, Mannheim, Germany).

Total RNA was extracted from transgenic plants using TRIzol reagent (Invitrogen, Carlsbad, CA, USA), and Northern blot analysis was conducted as described previously [6]. Membranes were hybridized with [^32^P]-dCTP-labeled probes specific for either the TYLCCNB-Y25 *βC1*.

### 4.7. Fluorometric GUS Assay

Leaves of four-week-old *N. benthamiana* plants were infiltrated with the *A. tumefaciens* harboring the various expression constructs fused to GUS marker gene, respectively. About 64 h after infiltration, GUS activity was measured as relative fluorescence units (RFU) per mg total protein per minute of 4-methyl umbelliferone (MU), produced by hydrolysis of 4-methyl umbelliferyl-β-d-glucuronide (MUG) [37]. The mean GUS activity from the pINT121 vector containing the *GUS* gene driven by the CaMV 35S promoter was considered to be 100% and was used to standardize the GUS activities in constructs driven by other promoters. The resulting data were analyzed using the Least Significant Difference (LSD) method implemented in the SPSS v12.0 software (SPSS, Chicago, IL, USA).

### 4.8. Fluorometric GFP Assay

Leaves of four-week-old *N. benthamiana* plants were infiltrated with the *A. tumefaciens* harboring the various expression constructs fused to *GFP* marker gene, respectively. About 64 h after infiltration, 1 cm^2^ leaf fragments were excised and GFP fluorescence was examined in epidermal cells by Confocal Laser Scanning Microscope (CLSM, Leica TCS SP5, Mannheim, Germany).

## Supplementary Materials

Supplementary materials can be found at http://www.mdpi.com/1999-4915/7/9/2853/s1.
