# Supplementary material for: Determinants of Disease Phenotype Differences Caused by Closely-Related Isolates of Begomovirus Betasatellites Inoculated with the Same Species of Helper Virus
Source: Viruses. 2015 Sep 14;7(9):4945–59. doi: 10.3390/v7092853 (PMC4584297; doi:10.3390/v7092853)
Supplement: Supplementary file 1 [file viruses-07-02853-s001.pdf]

# Supplementary Information

**Table S1.** Sequences of oligonucleotide primers used in PCR.

| Primer | Sequence (5′–3′)[a] | Position on satellite[b] (nt) |
|---|---|---|
| Primers used for construction of infectious clone of hybrid satellites | | |
| Y10βC1F | CGTATGCATAC**GTATTCATACATCTGAATTTGT** | 206–227 in Y10β |
| Y10βC1R | CCACAGCATA**AAACATGACTATCAAATACAAC** | 570–549 in Y10β |
| Y25βC1F | **CGTATATATAT**GTATTCATACACTTGAGTTTAT | 206–227 in Y25β |
| Y25βC1R | **CCACAACAAAT**AAACATGACAATCACATACAAT | 570–549 in Y25β |
| Y10βdC1F | GTATGTGATT**GTCATGTTTATTTGTTGTGG** | 562–581 in Y10β |
| Y10βdC1R | AACTCAAG**TGTATGAATACATATATATACGT** | 216–194 in Y10β |
| Y25βdC1F | **GTTGTATTTGATA**GTCATGTTTTATGCTGTGG | 562–580 in Y25β |
| Y25βdC1R | **AATTCAGA**TGTATGAATACGTATGCATACG | 216–195 in Y25β |
| Y10βPPF | TATTATGGCTAATAGA**GAAATGGAAAGAGGAGTAG** | 768–786 in Y10β |
| Y10βPPR | AATACTCTTTACACC**TTTTTACACATAATAATCCAGAT** | 767–745 in Y10β |
| Y25βPPF | **TTATTATGTGTAAAAA**GGTGTAAAGAGTATTATGAAAG | 768–789 in Y25β |
| Y25βPPR | **TTCTACTCCTCTTTCCATTTC**ACACCTCTATTAGCCATAAT | 772–753 in Y25β |
| Y10/25βC1F1 | ATGTCTATA**ACTTGTACCATATCTTCTTGCCT** | 298–320 in Y10β/Y25β |
| Y10/25βC1R1 | AGGCAA**GAAGATATGGTACAAGTTATAGACAT** | 314–289 in Y10β/Y25β |
| Y10/25βC1F2 | TTCTTCTTGGCTAAT**GCTGGTGACTTTGTTGATG** | 454–472 in Y10β/Y25β |
| Y10/25βC1R2 | CATCAACAAA**GTCACCAGCATTAGCCAAGAAGAA** | 462–439 in Y10β/Y25β |

**Table S1.** *Cont.*

| Primer | Sequence (5′–3′)[a] | Position on satellite[b] (nt) |
|---|---|---|
| Primers used for amplification of promoter fragments | | |
| Y25βp-F | CGAATTCAAGCTTATACGTATGCATACGTATTC (*Eco*RI/*Hin*dIII) | 211–192 in Y25β |
| Y25βp1-F | CGAATTCAAGCTTCCTATCTCCACTAATATATATAT (*Eco*RI/*Hin*dIII) | 687–665 in Y25β |
| Y25βp2-F | CGAATTCAAGCTTACACCTCTATTAGCCATAAT (*Eco*RI/*Hin*dIII) | 772–753 in Y25β |
| Y25βp3-F | CGAATTCAAGCTTGTTGCATTTGCTTGTTTCAC (*Eco*RI/*Hin*dIII) | 931–912 in Y25β |
| Y25βp4-F | CGAATTCAAGCTTAACCGTTGGAACGATTTTAC (*Eco*RI/*Hin*dIII) | 1007–988 in Y25β |
| Y25βp5-F | CGAATTCAAGCTTGCAAATTGATGACCGGAAG (*Eco*RI/*Hin*dIII) | 1240–1222 in Y25β |
| Y25βp6-F | CGAATTCAAGCTTTTTCTTCTTCTATGTGGGAC (*Eco*RI/*Hin*dIII) | 55–36 in Y25β |
| Y25βp-R | CGAGCTCGGATCCGTTTTATGCTGTGGATTATAC (*Sac*I/*Bam*HI) | 587–567 in Y25β |
| Primers used for construction of transgenic expression vectors | | |
| Y25βC1-F | GGATCCATGACAATCACATACAATAACAT (*Bam*HI) | 566–544 in Y25β |
| Y25βC1-R | GCGTCGACTCATACACTTGAGTTTATAAATAC (*Sal*I) | 210–233 in Y25β |

[a] The sequences in bold are complementary to TYLCCNB-Y10, and the underlined restriction sites are indicated in the brackets, respectively.[b]Y10β, TYLCCNB-Y10; Y25β, TYLCCNB-Y25.
